# A Novel Approach for Blast-Induced Flyrock Prediction Based on Imperialist Competitive Algorithm and Artificial Neural Network

**DOI:** 10.1155/2014/643715

**Published:** 2014-07-22

**Authors:** Aminaton Marto, Mohsen Hajihassani, Danial Jahed Armaghani, Edy Tonnizam Mohamad, Ahmad Mahir Makhtar

**Affiliations:** ^1^Department of Geotechnics and Transportation, Universiti Teknologi Malaysia (UTM), 81310 Skudai, Johor, Malaysia; ^2^Construction Research Alliance, Universiti Teknologi Malaysia (UTM), 81310 Skudai, Johor, Malaysia; ^3^Department of Structures and Materials, Universiti Teknologi Malaysia (UTM), 81310 Skudai, Johor, Malaysia

## Abstract

Flyrock is one of the major disturbances induced by blasting which may cause severe damage to nearby structures. This phenomenon has to be precisely predicted and subsequently controlled through the changing in the blast design to minimize potential risk of blasting. The scope of this study is to predict flyrock induced by blasting through a novel approach based on the combination of imperialist competitive algorithm (ICA) and artificial neural network (ANN). For this purpose, the parameters of 113 blasting operations were accurately recorded and flyrock distances were measured for each operation. By applying the sensitivity analysis, maximum charge per delay and powder factor were determined as the most influential parameters on flyrock. In the light of this analysis, two new empirical predictors were developed to predict flyrock distance. For a comparison purpose, a predeveloped backpropagation (BP) ANN was developed and the results were compared with those of the proposed ICA-ANN model and empirical predictors. The results clearly showed the superiority of the proposed ICA-ANN model in comparison with the proposed BP-ANN model and empirical approaches.

## 1. Introduction

Blasting is the process of using explosives to excavate, break down, or remove the rock. It is the most frequently used method for fragmentation of rock mass in smining, quarrying, and civil engineering applications such as dam or road construction. Rock is fragmented into smaller pieces in different mining operations such as quarrying or into large blocks for some civil engineering applications [1]. In quarry blasting, only 20 to 30 percent of the produced energy is utilized to fragment and move the rock mass [2]. The remained energy is wasted to create unwanted environmental impacts, for example, air-overpressure, ground vibration, flyrock, dust, and back-break [3]. Flyrock is defined as the excessive random throw of rock fragments from a blast that can travel distances beyond the blast safety area [4, 5]. This phenomenon of the blasting operation can result in human injuries, fatalities, and structural damage [4, 6].

Various empirical relationships have been established to predict flyrock resulted from blasting [7–10]. Nevertheless, the existing empirical methods only consider limited numbers of effective parameters on flyrock distance, whereas this phenomenon is also affected by other parameters such as blast geometry and geological conditions [11]. As a result, the empirical methods are not accurate enough in many cases, even though prediction of the exact values of the flyrock distance is crucial to estimate the blast safety area [12]. Apart from that, statistical methods such as multiple regression for flyrock prediction have drawn attention mainly due to their ease of use [13]. However, the implementation of the regression prediction methods is not reliable if new available data are different from the original ones as the form of the obtained equation needs to be updated. Meanwhile, the feasibility of artificial intelligence techniques such as artificial neural networks (ANNs) in predicting the flyrock distance has been reported in many studies [14–16].

ANNs are one of the most dynamic areas of research in advanced and diverse applications of science and engineering. Although ANNs are able to directly map input to output patterns and utilize all influential parameters in prediction of flyrock, there are still some limitations, the slow rate of learning and getting trapped in local minima [17, 18]. To overcome these shortcomings, employing imperialist competitive algorithm (ICA) is of advantage. ICA is a population-based evolutionary algorithm inspired by human being's sociopolitical evolution [19]. This algorithm has been successfully utilized in the various optimization engineering problems [20–24]. This paper presents a hybrid ICA-ANN predictive model for flyrock prediction in Putri Wangsa quarry in Johor, Malaysia. For the sake of comparison, the results of the developed model are compared to the results of an empirical equation and multivariate regression analysis.

## 2. Flyrock and Effective Parameters

Flyrock is unwanted rock fragments thrown during bench blasting in mines and civil constructions [5]. Flyrock, which is propelled rock fragments by energy of explosive beyond the blast zone, is one of the undesirable environmental impacts of blasting operations [25]. In flyrock mechanism, there is an affective relationship among explosive energy distribution, rock mass mechanical strength, and charge confinement. According to Bajpayee et al. [26], any mismatch between these parameters can produce flyrock. When this happens, much of the explosive energy is used to throw the rock rather than produce fragmented rock [6].

There are numerous causes for flyrock occurrence ranging from abnormalities in blast pattern or their implementation, explosive use, and known or unknown rock mass conditions [27–29]. There are several researches that report the effects of abovementioned factors on flyrock distance. Fletcher and D'Andrea [11] explained that excessive flyrock gets projected beyond the blast safety area and is created due to much explosive energy for the amount of burden, insufficient stemming, and venting of explosive energy through a weak plane. According to Bhandari [1] and Hemphill [30], inadequate burden and spacing, inadequate stemming, inaccurate drilling, overloaded holes, excessive powder factor, and unfavorable geological conditions are the main causes of flyrock.

Several empirical equations have been established by some researchers to predict flyrock distance. Lundborg et al. [7] suggested an empirical equation based on hole and rock diameters to predict flyrock distance as follows:

(1) $$\begin{array}{c} L_{m}=260\times D^{2/3},\\ T_{b}=0.1\times D^{2/3}, \end{array}$$

in which *L*_*m*_ is the maximum rock throw in meters, *D* is hole diameter in inches, and *T*_*b*_ is the size of rock fragment in meters. Gupta [10] proposed an empirical equation for prediction of flyrock based on stemming length and burden, as given below:

(2) $$\begin{array}{c} L=155.2\times d^{-1.37}, \end{array}$$

where *L* is the ratio of length of stemming column to burden and *d* is the distance travelled by the flying fragments in meters. McKenzie [31] suggested equations to predict the maximum range of flyrock and the particle size (achieving the maximum range) for blasts of varying rock density, hole diameter, explosive density, and state of confinement. He demonstrated that the flyrock travel range is based on hole diameter, shape factor, and size of rock fragment that achieves maximum projection distance in terms of rock density and shape factor. This study was very significant in defining the danger zone of blasting.

Apart from empirical methods, many researchers have been working on prediction of flyrock distance using soft computing techniques. Monjezi et al. [32] used ANN to predict flyrock that resulted from blasting operations. They employed 192 datasets to train and evaluate ANN simulations and showed the high performance of ANN model to predict flyrock. Based on their results, it was found that blast ability index, charge per delay, hole diameter, stemming length, and powder factor are the most effective parameters on flyrock distance. Rezaei et al. [13] applied a fuzzy interface system (FIS) to predict flyrock and compared the FIS results with conventional statistical approaches and indicated that the efficiency of the developed FIS model is much better than that of statistical models. Ghasemi et al. [16] developed two predictive models based on ANN and FIS models in predicting flyrock distance and showed that both models are able to predict flyrock distance in which the FIS model yielded higher performance compared to the ANN model. Monjezi et al. [33] used neurogenetic model to predict flyrock and back-break and found that the stemming length and powder factor are the most influential parameters on flyrock. In other study of flyrock prediction, Jahed Armaghani et al. [12] predicted flyrock distance using hybrid particle swarm optimization (PSO) and ANN. They used PSO to improve the performance of ANN in predicting flyrock that resulted from blasting operations in granite quarry sites. Finally, their results indicated the applicability of the proposed model to predict flyrock distance.

## 3. Case Study

The data used in this study was collected from the Putri Wangsa quarry in Johor, Malaysia. The quarry lies geographically in latitude 1°35′32′′N and longitude 103°48′4′′E and is located at north of Johor. This quarry produces aggregates for various construction applications with capacity of 40000–50000 tonnes per month. 10 to 12 blasting operations were conducted monthly in the quarry depending on the weather condition. A complete range of mass weathering grades from fresh to completely weathered rock was observed [34]. Blasting parameters such as burden, spacing, stemming length, hole depth, and number of holes were recorded for each blasting. Besides, for each blasting, rock density and Schmidt hammer rebound value were measured as strength parameters of rock mass. Diameter of blast-holes used in this quarry was 115 mm. Ammonium nitrate and fuel oil (ANFO) and dynamite were used as the main explosive material and initiation, respectively. The blast-holes were stemmed using fine gravels. To measure the flyrock distance in the Putri Wangsa, the bench surface was colored and two video cameras were placed to record the flyrock projection. After each blasting, the relevant videos were reviewed to find the locations of the traveled rocks.

## 4. Model Development for Flyrock Prediction

### 4.1. Artificial Neural Network

An artificial neural network is a mathematical model which works on the basis of simulating the cortical configuration of the human brain. In other words, an ANN is a flexible nonlinear function approximation that comprehends a relationship between desired input and output data. ANNs require training to learn and consequently map a relationship from the data. The ability of ANNs to learn from samples and to improve their performance through learning is the property that makes them different from other networks. This ability comes from training algorithm. The details of different ANN methods and training algorithm can be found in Simpson [35] and Galushkin [36].

An interconnected group of artificial neurons forms the ANN structure. An artificial neuron is a simple processor which is connected to other neurons. The artificial neurons get the data and implement simple processing on the received data. Subsequently, the artificial neurons pass the processed information to other neurons through an activation function that usually is a nonlinear function. By this process, a computational model is created for the information processing. According to Fausett [37], artificial neurons have been developed as generalizations of mathematical models of biological neurons on the foundation of the following assumptions:

- neurons which are simple elements conduct data processing;
- connection links transfer the data between neurons;
- a weight is assigned to each connection link which is multiplied in transmitted signal;
- an activation function is used by each neuron to determine its output signal.

McCulloch and Pitts [38] introduced the earliest neuron called “Threshold Logic Unit” which was a linear function. Nevertheless, the first ANN was developed by Rosenblatt [39], called the “perceptron,” based on the work of McCulloch and Pitts [38]. A set of parallel interconnected processing units named nodes or neurons forms the basis of an ANN. At the final step of data processing, the network output is verified with the actual values and error correction is performed. In feed-forward ANNs, the neurons are usually classified into several layers. Using the connections, a signal moves throughout the input to the output layers. Multilayer perceptron (MLP) is the most well-known type of feed-forward ANN [32, 33].

### 4.2. Imperialist Competitive Algorithm

Imperialist competitive algorithm (ICA) is a computational method which is utilized to solve different types of optimization problems [19]. ICA, as a new sociopolitically motivated global search algorithm, indicated great performance in the convergence rate [19, 40–42]. Similar to most of the methods in the area of evolutionary computation, ICA does not require the gradient of the function in its optimization process.

**Figure 1:**
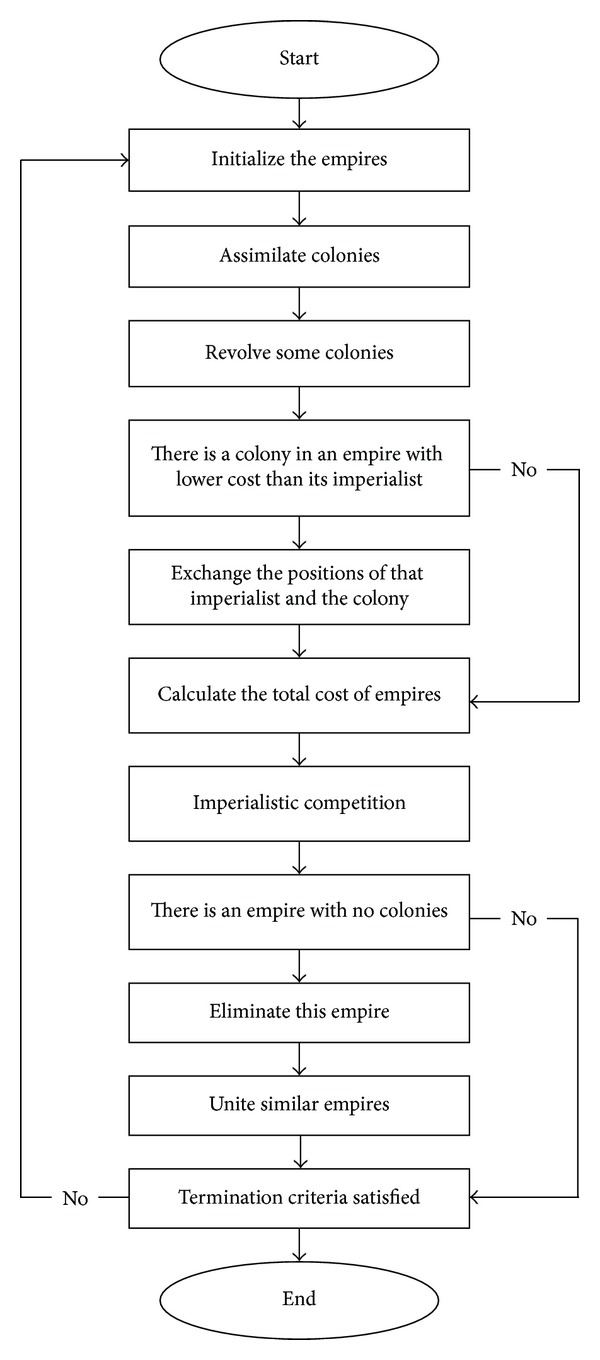
Flowchart of the ICA.

Figure 1 shows the flowchart of the ICA. According to this figure, the optimization process starts with producing the population. In this algorithm, each particle of the population is called a “*country.*” The countries are divided into two sections; the best countries (countries with the minimum cost) are considered the “*imperialists*” and the rest of the countries form the “*colonies.*” All colonies are distributed among the existing imperialists on the basis of their power. The combination of each imperialist together with its colonies forms an empire. Following the establishment of initial empires, the colonies move toward their relevant imperialists and simulate the assimilation policy of imperialist states. The following steps describe the ICA optimization procedure.

#### 4.2.1. Establishment of Initial Empires

The ICA optimization procedure starts with initializing the individuals which are called countries. In a multivariate optimization problem, a country consists of 1 × *N*_var_ array. This array is defined as follows:

(3) $$\begin{array}{c} \text{Country}=\left[P_{1},P_{2},P_{3},\ldots ,P_{N\;\text{variable}}\right], \end{array}$$

in which *P*_*i*_s are the parameters which need to be optimized. In a country, each parameter can be considered as a sociopolitical characteristic such as culture and language, in which ICA makes an attempt to find the best combination of these characteristics. The cost function of each country *f*(Country) is determined as follows:

(4) $$\begin{array}{c} f\left(\text{Country}\right)=\left[P_{1},P_{2},P_{3},\ldots ,P_{N\;\text{variable}}\right]. \end{array}$$

The procedure of ICA optimization starts with initializing of countries of size *N*_country_ and selecting the most powerful countries as the imperialists (*N*_imperialist_). The remaining countries are considered as the colonies (*N*_colony_). The colonies are divided among imperialists based on their power to generate the initial empires. Therefore, the normalized cost of an imperialist is defined as follows:

(5) $$\begin{array}{c} C_{n}=c_{n}-\underset{i}{\max}\left\{c_{i}\right\}, \end{array}$$

in which *c*_*n*_ is the cost of the *n*th imperialist and *C*_*n*_ is its normalized cost. The normalized power of each imperialist is defined as follows:

(6) $$\begin{array}{c} p_{n}=\left|\frac{C_{n}}{\sum_{i=1}^{N_{\text{imp}}}C_{i}}\right|. \end{array}$$

The number of initial colonies for each empire is obtained by

(7) $$\begin{array}{c} \text{N}.\text{C}._{n}=\text{round}\left\{p_{n}\cdot N_{\text{col}}\right\} \end{array}$$

in which N.C._*n*_ is the initial number of colonies of the *n*th empire and *N*_col_ is the total number of initial colonies.

To distribute the colonies among imperialists, N.C._*n*_ of the colonies is accidentally selected and yielded to the *n*th imperialist and therefore generates the *n*th empire.

#### 4.2.2. Assimilation, Revolution, and Uniting

**Figure 2:**
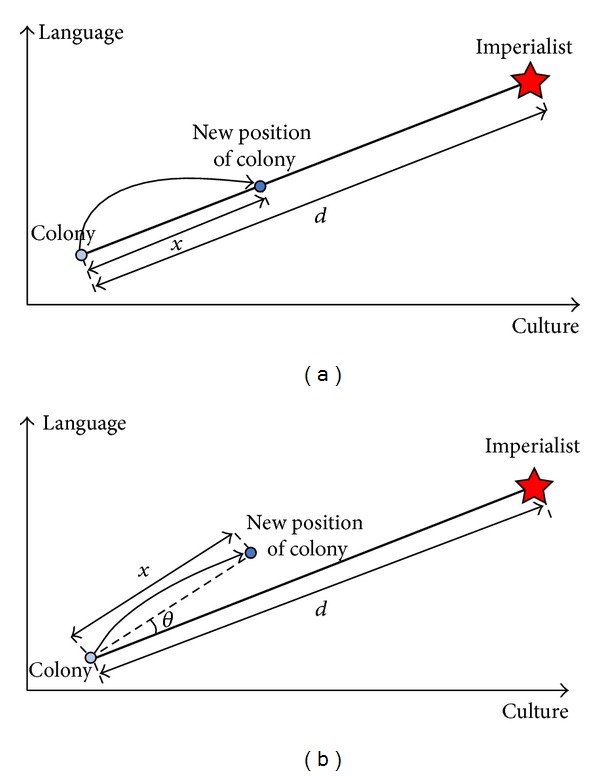
Movement of colonies (a) toward their relevant imperialist and (b) in a randomly deviated direction [19].

In this step, assimilation and revolution are conducted. Assimilation is the movement of colonies toward the imperialists where imperialists try to absorb their colonies and make them a part of themselves. This process is simulated by moving all colonies toward the imperialist along different optimization axis.  Figure 2(a) illustrates the movement of a colony toward its relevant imperialist by *x* units. The parameter *x* is determined as follows:

(8) $$\begin{array}{c} x\sim U\left(0,\beta \times d\right), \end{array}$$

where *d* is the distance between the colony and the imperialist and *β* is a number greater than 1. In assimilation process, the movement direction is not essentially a vector from the colony to the imperialist. Hence, to increase the searching ability around the imperialist, a random amount of deviation (*θ*) is added to the movement direction, as shown in Figure 2(b). *θ* is a parameter with uniform distribution and is obtained as follows:

(9) $$\begin{array}{c} \theta \sim U\left(-\gamma ,\gamma \right), \end{array}$$

in which *γ* is a parameter that adjusts the deviation from the original direction.

**Figure 3:**
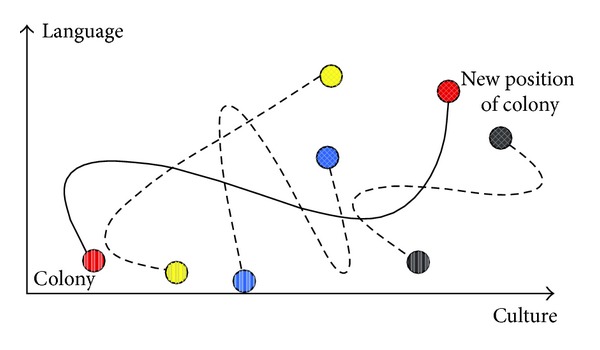
Sudden changes in sociopolitical characteristics of a country [19].

Following the assimilation, revolution happens. Revolution is defined as changes in the power and structure that happen quickly. In ICA optimization process, revolution makes a sudden change in the sociopolitical characteristics of a country. This action increases the exploration of the algorithm and impedes the quick convergence of countries to local minima.  Figure 3 illustrates the revolution in sociopolitical characteristics of a country. Throughout the moving of colonies toward the imperialist, a colony may obtain a position with lower cost compared to its imperialist. In this case, the positions of the colony and the imperialist are altered and ICA procedure will be continued by the new imperialist in the new position.

Uniting similar empires happens when the distance between two imperialists becomes lesser than threshold distance. On this occasion, these imperialists are united and a new empire is formed. The total power of an empire is obtained as follows:

(10) T.C.n=Cost(imperialistn) +ξ mean{cost(colonies  of  empiren)},

in which T.C._*n*_ is the total cost of the *n*th empire and *ξ* is a positive small number. The value of 0.1 for *ξ* has shown good results in most of the implementations [19].

#### 4.2.3. Imperialistic Competition

**Figure 4:**
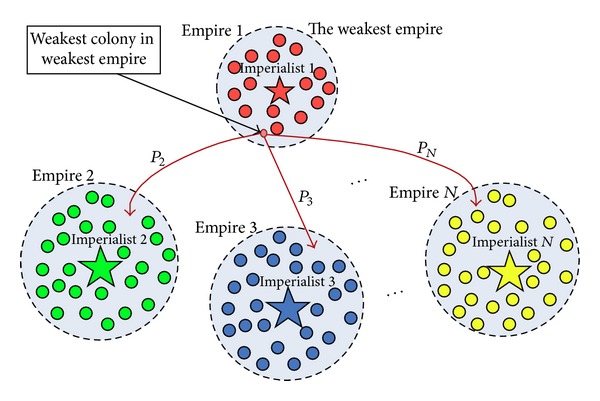
Imperialistic competition [19].

In ICA optimization procedure, all empires make an attempt to possess the colonies of other empires. In ICA terminology, this action is called “imperialistic competition” which is the final optimization step. In this regard, the power of the weaker empires is decreased and the power of more powerful empire is gradually increased. The imperialistic competition is shown in Figure 4.

To start the imperialistic competition, the weakest colony of the weakest empire is selected and subsequently the possession probability of each empire is found. The possession probability of an empire (*P*_*P*_) is related to the total power of the empire. The normalized total cost of an empire is obtained as follows:

(11) $$\begin{array}{c} \text{N}.\text{T}.\text{C}._{n}=\text{T}.\text{C}._{n}-\max\left\{\text{T}.\text{C}._{i}\right\}\;, \end{array}$$

in which T.C._*n*_ and N.T.C_*n*_ are the total cost and the normalized total cost of the *n*th empire, respectively. The possession probability of each empire is obtained as follows:

(12) $$\begin{array}{c} P_{P_{n}}=\left|\frac{\text{N}.\text{T}.{\text{C}.}_{n}}{\sum_{i=1}^{N}\text{N}.\text{T}.{\text{C}.}_{i}}\right|, \end{array}$$

in which *P*_*P*_*n*__ is the possession probability. Vector *P* is created to distribute the colonies among empires as follows:

(13) $$\begin{array}{c} P=\left[P_{P_{1}},P_{P_{2}},P_{P_{3}},\ldots ,P_{P_{N}}\right]. \end{array}$$

Subsequently, vector *R* with uniform distributed random elements is created as follows:

(14) $$\begin{array}{c} R=\left[r_{1},r_{2},r_{3},\ldots ,r_{N}\right]\;r_{1},r_{2},r_{3},\ldots ,r_{N}\sim U\left(0,1\right), \end{array}$$

in which *R* is a chromatic vector with the same size as *P*. Then vector *D* is created by subtracting vector *R* from vector *P* as follows:

(15) $$\begin{array}{c} D=P-R=\left[P_{P_{1}}-r_{1},P_{P_{2}}-r_{2},P_{P_{3}}-r_{3},\ldots ,P_{P_{N}}-r_{N}\right]. \end{array}$$

Referring to vector *D*, the colonies are handed to an empire whose relevant index in *D* is maximized. Continuing the aforementioned steps may lead to finding the global minimum.

### 4.3. Model Development

Various optimization algorithms have been utilized for training ANNs to achieve a set of weights and biases that minimize the error functions. Here, ICA was used to determine the optimum weights and biases of a feed-forward MLP ANN in order to obtain the best correlation in flyrock prediction. Therefore, a three-layered ANN that consisted of an input layer, a hidden layer, and one output layer was employed. The most influential parameters on flyrock were utilized as input parameters and flyrock distance was set as the output parameter. The input and output parameters used in the modelling are tabulated in Table 1.

**Figure 5:**
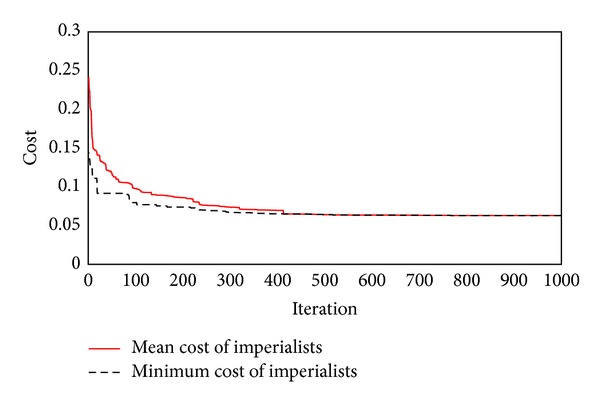
Mean and minimum cost of all imperialists in various iterations for the selected model.

Determining the optimal weights and biases of ANN can be formulated as a global search problem in ICA. In this regard, a Matlab code was prepared to train ANN using ICA and the weights and biases of ANN were considered as the variables of optimization problem. A criterion is required to evaluate the modelling performance. Therefore, root mean square error (RMSE) was proposed as the cost function of ICA optimization algorithm, whereas the aim of modelling is to minimize the cost function. In ICA optimization procedure, the problem's solution can be obtained by choosing adequate amount of the number of countries and imperialists. Therefore, 20 models with different number of countries and imperialists were employed to determine the optimum number of countries and imperialists. The results of modelling are tabulated in Table 2. According to this table, with 56 countries and 9 imperialists, the best results were obtained among all models and therefore this model was selected to be used in flyrock prediction.  Figure 5 illustrates the minimum and mean costs of all imperialists in the selected model.

To obtain the best results of ANN trained by ICA, it is essential to find the optimum network architecture which is the placement of various components of a network. ICA can only adjust the weights and biases of an ANN to minimize the learning error and cannot determine the optimum network architecture. According to Hornik et al. [43], a network with one hidden layer can approximate any continuous function. Consequently, several networks with one hidden layer with different number of nodes were trained and tested to determine the optimum network architecture. Coefficient of determination (*R* ^2^) and RMSE (16) were considered the criteria to evaluate the accuracy of each model. Consider

(16) $$\begin{array}{c} R^{2}=1-\frac{\sum_{i=1}^{N}{\left(y-y^{\prime }\right)}^{2}}{\sum_{i=1}^{N}{\left(y-\tilde{y}\right)}^{2}},\\ \text{RMSR}=\sqrt{\frac{1}{N}\overset{N}{\underset{i=1}{\sum }}{\left(y-y^{\prime }\right)}^{2},} \end{array}$$

where *y* and *y*′ are the measured and predicted values, respectively. $\tilde{y}$ represents the mean of *y* values and *N* is the total number of data.

**Figure 6:**
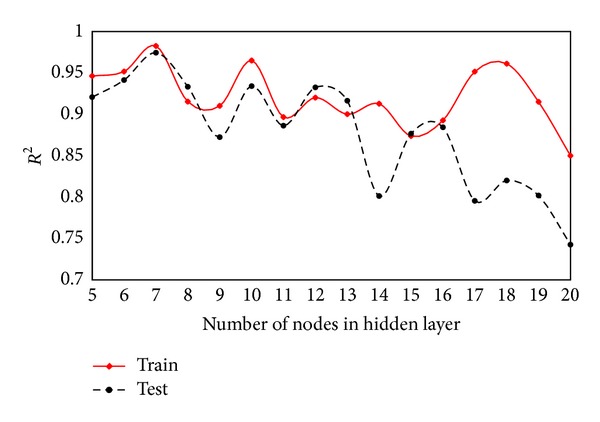
*R* ^2^ of various models for training and testing datasets.

**Figure 7:**
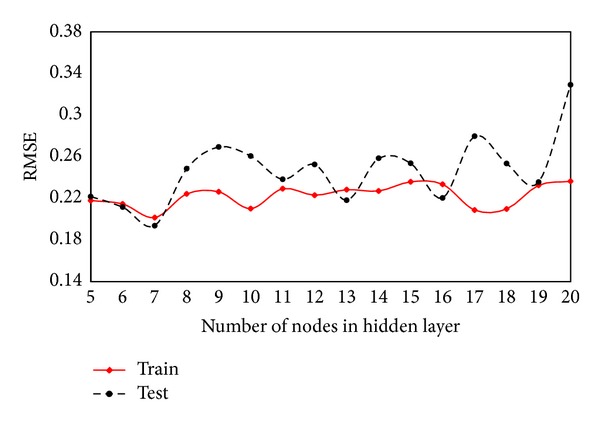
RMSE of various models for training and testing datasets.

In the modelling process, 80% of data were used for training and the rest for testing. The results of analyses for training and testing datasets for various models are shown in Figures 6 and 7, respectively. As in these figures, the model with seven nodes in the hidden layer shows the best results among all models and therefore was selected to be used in predicting flyrock distance.

## 5. Multivariate Regression

Multivariate regression analysis (MRA) can be used to obtain the best-fit equation when there is more than one input parameter. The MRA equation takes the form of *y* = *b*_1_ *x*_1_ + *b*_2_ *x*_2_ + *b*_*n*_ *x*_*n*_ + *c*, where {*b*_1_, *b*_2_,…, *b*_*n*_} are the regression coefficients. The parameter *c* is a constant value of *y* parameter, when all the input variables are zero. Ceryan et al. [44] mentioned that as long as independent parameters have acceptable correlation or determination with output, they can be selected as inputs in predictive models.

In order to propose a new equation to predict flyrock distance, a MRA model was applied using the same inputs in ICA-ANN model (see Table 1). The statistical software package SPSS (18.0) was used for analysis. The obtained equation using MVR analysis is shown in (17). More details on the statistical information of the proposed equation for flyrock prediction can be found in Table 3. Consider

(17) Flyrock=−11.873A−10.296B+136.128D −14.218E+0.282F+1.562G+9.665.

## 6. Empirical Model Development

A sensitivity analysis was performed to establish empirical equation for flyrock prediction. For this purpose, the cosine amplitude method was used. To perform this technique, all data pairs were utilized to build a data array *X* as follows:

(18) $$\begin{array}{c} X=\left\{x_{1},x_{2},x_{3},\ldots ,x_{i},\ldots ,x_{n}\right\}. \end{array}$$

The variable *x*_*i*_ in the array *X* is a length vector of m as

(19) $$\begin{array}{c} x_{i}=\left\{x_{i1},x_{i2},x_{i3},\ldots ,x_{im}\right\}. \end{array}$$

The following equation presents the strength of the relation (*r*_*ij*_) between the datasets *X*_*i*_ and *X*_*j*_:

(20) $$\begin{array}{c} r_{ij}=\frac{\sum_{k=1}^{m}x_{ik}x_{jk}}{\sqrt{\sum_{k=1}^{m}x_{ik}^{2}\sum_{k=1}^{m}x_{jk}^{2}}}. \end{array}$$

**Figure 8:**
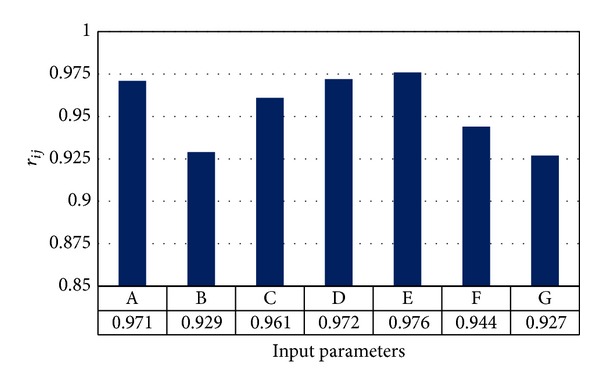
Strengths of relation between inputs and flyrock distance.

Figure 8 shows the strengths of the relations (*r*_*ij*_ values) between flyrock distance and input parameters. As shown in this figure, powder factor and maximum charge per delay are the most influential parameters on flyrock.

**Figure 9:**
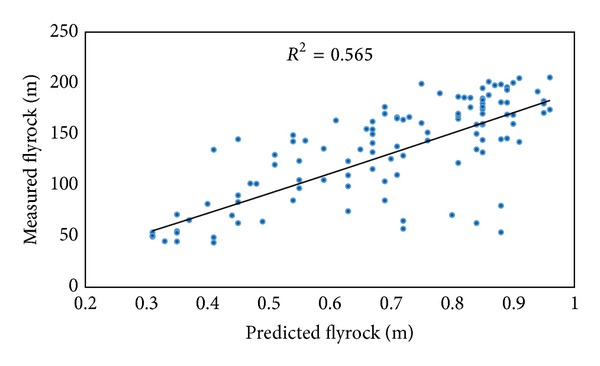
*R* ^2^ of proposed flyrock equation based on powder factor.

**Figure 10:**
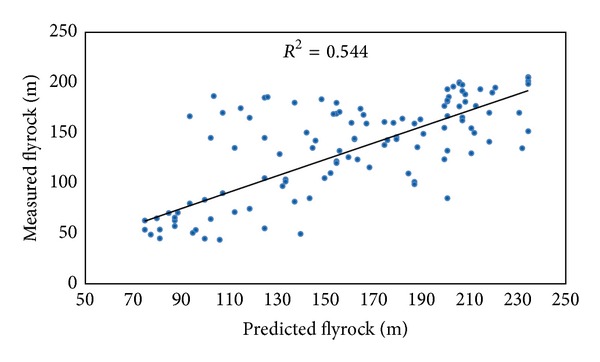
*R* ^2^ of proposed flyrock equation based on maximum charge per delay.

By using these parameters, two power empirical equations were developed. *R* ^2^ of these equations based on powder factor and maximum charge per delay are shown in Figures 9 and 10, respectively. It should be mentioned that other equation models such as linear, exponential, and logarithmic were also examined and power equations which showed the higher performance (in terms of *R* ^2^ and RMSE) compared to other models were selected.  Table 4 shows the proposed equations and their performance for flyrock prediction.

## 7. Results and Discussion

**Figure 11:**
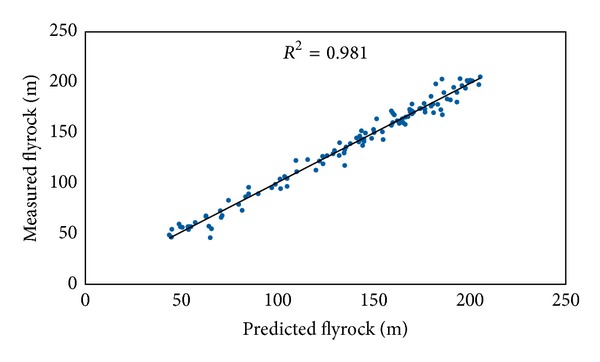
*R* ^2^ of ICA-ANN model in predicting flyrock distance.

**Figure 12:**
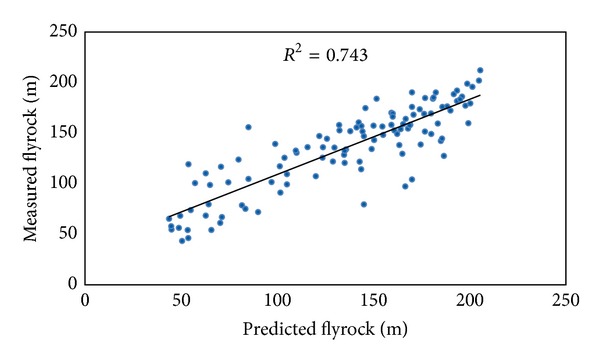
*R* ^2^ of MRA model in predicting flyrock distance.

In this study ICA-ANN and MRA models were developed to predict flyrock distance. These models were constructed using seven inputs (hole depth, stemming, burden to spacing, maximum charge per delay, powder factor, rock density, and Schmidt hammer rebound number) and one output. The graphs of predicted flyrock using ICA-ANN and MRA techniques against the measured flyrock are displayed in Figures 11 and 12, respectively.

**Figure 13:**
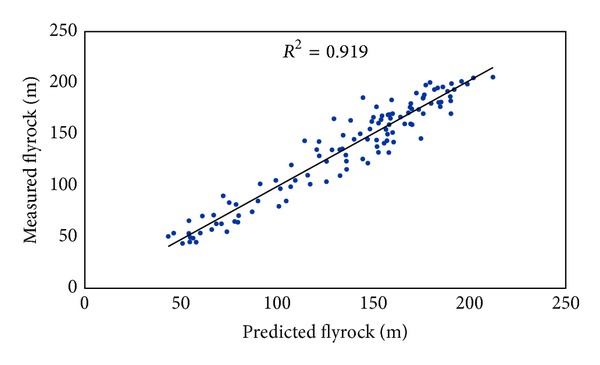
*R* ^2^ of BP-ANN model in predicting flyrock distance.

**Figure 14:**
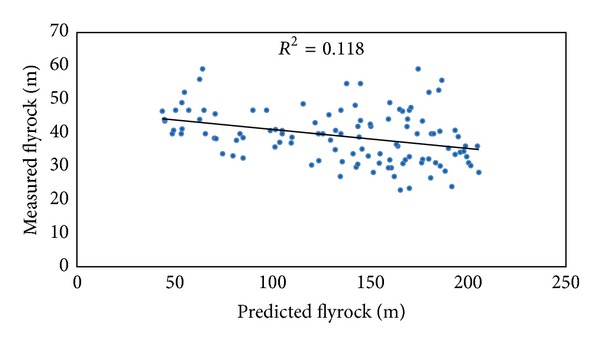
*R* ^2^ of (2) in predicting flyrock distance.

It is desirable to increase the accuracy and applicability of ANN for the prediction of flyrock induced by blasting by means of changing the learning process. A predeveloped backpropagation (BP) ANN was utilized to predict flyrock in order to make a comparison with the ICA-ANN performance. Similar to the ICA-ANN models, several BP-ANN models with different architectures were constructed by using the same input parameters. The datasets were divided into two subsets; that is, 80% of the datasets were set for training purpose and 20% used for testing the network performance. Finally, a BP-ANN model with one hidden layer and 8 neurons in the hidden layer was selected as the best ANN model. It should be noted that in the suggested ANN model the momentum coefficient and learning rate were set to be 0.9 and 0.05, respectively. The graph of predicted flyrock using the proposed BP-ANN model against the measured flyrock is shown in Figure 13. In addition, to check the accuracy of the predeveloped empirical equations, (2) was selected for prediction of flyrock using the data of Putri Wangsa quarry.  Figure 14 shows the predicted flyrock values using (2) versus the measured one.

For the sake of comparison, the performance indices of the predictive models are tabulated in Table 5. The obtained results by ICA-ANN, BP-ANN, and MRA models as well as empirical equations reveal that the proposed ICA-ANN model produced higher performance in predicting flyrock distance among all methods.

## 8. Conclusion

A novel approach based on the combination of ICA and ANN was developed to predict flyrock induced by blasting. In this regard, 113 blasting operations in Putri Wangsa quarry site in Malaysia were precisely recorded and collected data were utilized to train the ICA-ANN model. The most influential parameters on flyrock including hole depth, burden to spacing ratio, stemming length, maximum charge per delay, powder factor, rock density, and Schmidt hammer rebound number were considered as input parameters, whereas the flyrock distances were assigned as the output parameter. Several models were examined using the collected data to determine the optimum ICA-ANN model and finally an optimum model was proposed to be used in flyrock prediction. The results demonstrated that the proposed ICA-ANN model is able to predict flyrock distance with high degree of accuracy. For a comparison purpose, a predeveloped BP-ANN model was developed and the results were compared with the obtained results of proposed ICA-ANN model. By means of sensitivity analysis, powder factor and maximum charge per delay were determined as the most influential parameters on flyrock. By utilizing these parameters as well as MRA, two empirical predictors were developed to predict flyrock distance. These predictors provide very quick and simple prediction, whereas the proposed ICA-ANN model exhibited higher prediction performance compared to other methods.

## Figures and Tables

**Table 1:** Input and output parameters used in the predictive model.

Parameter	Category	Unit	Symbol	Minimum	Maximum	Average
Hole depth	Input	(m)	A	7.5	22	15.434
Burden to spacing	Input	—	B	0.410	0.913	0.763
Stemming length	Input	(m)	C	1.5	3.5	2.632
Maximum charge per delay	Input	(Kg)	D	74.8	234.3	159.6
Powder factor	Input	(Kg/m^3^)	E	0.31	0.96	0.7
Rock density	Input	(g/cm^3^)	F	2.15	2.86	2.574
Schmidt hammer rebound number	Input	—	G	15	44	32.611
Flyrock distance	Output	(m)	H	43.7	205.5	135.75

**Table 2:** Mean and minimum cost of imperialists for different models.

Model number	Country/imperialist	Number of countries	Number of imperialists	Mean cost of imperialists	Minimum cost of imperialists	Coefficient of determination	Root mean square error
1	2.7	8	3	0.073	0.073	0.880	0.241
2	4.0	16	4	0.074	0.074	0.882	0.240
3	4.8	24	5	0.078	0.078	0.856	0.250
4	5.3	32	6	0.092	0.092	0.842	0.255
5	5.7	40	7	0.075	0.073	0.878	0.241
6	6.0	48	8	0.079	0.070	0.896	0.235
7	6.2	56	9	0.063	0.063	0.924	0.224
8	6.4	64	10	0.080	0.073	0.878	0.241
9	6.5	72	11	0.075	0.069	0.899	0.233
10	6.7	80	12	0.077	0.071	0.892	0.236
11	6.8	88	13	0.079	0.074	0.876	0.242
12	6.9	96	14	0.083	0.063	0.922	0.224
13	6.9	104	15	0.083	0.064	0.920	0.225
14	7.0	112	16	0.077	0.070	0.894	0.235
15	7.1	120	17	0.082	0.072	0.886	0.238
16	7.1	128	18	0.077	0.065	0.913	0.228
17	7.2	136	19	0.078	0.063	0.931	0.225
18	7.2	144	20	0.078	0.068	0.899	0.234
19	7.2	152	21	0.079	0.067	0.902	0.232
20	7.3	160	22	0.075	0.067	0.906	0.231

**Table 3:** Statistical information for developed predictive model.

Independent variable	Coefficients	St. error	*t*-value	*P* value
Constant	9.665	53.718	0.179	0.857
A	−11.872	8.993	−1.320	0.189
B	−10.296	20.550	−0.501	0.617
C	0	0	65535	—
D	136.127	14.095	9.657	—
E	−14.218	27.777	−0.511	0.609
F	0.282	0.747	0.377	0.706
G	1.562	0.812	1.922	0.057

**Table 4:** Proposed equations and their performance.

Model input	Equation	Model performance	
		*R* ^2^	RMSE
Powder factor	Flyrock = 191.11∗*E* ^1.059^	0.565	31.935
Maximum charge per delay	Flyrock = 0.883∗*D* ^0.986^	0.544	34.095

**Table 5:** Performance indices of the predictive models.

Predictive model	Performance indices	
	*R* ^2^	RMSE
ICA-ANN	0.981	6.582
BP-ANN	0.919	13.478
MRA	0.743	23.877
Empirical	0.118	109.064
